# Bioresorbable Chitosan-Based Bone Regeneration Scaffold Using Various Bioceramics and the Alteration of Photoinitiator Concentration in an Extended UV Photocrosslinking Reaction

**DOI:** 10.3390/gels8110696

**Published:** 2022-10-28

**Authors:** Farah Alwani Azaman, Keran Zhou, María del Mar Blanes-Martínez, Margaret Brennan Fournet, Declan M. Devine

**Affiliations:** 1 PRISM Research Institute, Technological University of the Shannon, Midlands Midwest, Athlone Main Campus, N37 HD68 Athlone, Ireland; 2 Department of Chemical and Nuclear Engineering, Universitat Politècnica de València, 46022 Valencia, Spain

**Keywords:** chitosan, hydroxyapatite, biodegradation, photoinitiator, photopolymerisation, crosslinking

## Abstract

Bone tissue engineering (BTE) is an ongoing field of research based on clinical needs to treat delayed and non-union long bone fractures. An ideal tissue engineering scaffold should have a biodegradability property matching the rate of new bone turnover, be non-toxic, have good mechanical properties, and mimic the natural extracellular matrix to induce bone regeneration. In this study, biodegradable chitosan (CS) scaffolds were prepared with combinations of bioactive ceramics, namely hydroxyapatite (HAp), tricalcium phosphate-α (TCP- α), and fluorapatite (FAp), with a fixed concentration of benzophenone photoinitiator (50 µL of 0.1% (*w/v*)) and crosslinked using a UV curing system. The efficacy of the one-step crosslinking reaction was assessed using swelling and compression testing, SEM and FTIR analysis, and biodegradation studies in simulated body fluid. Results indicate that the scaffolds had comparable mechanical properties, which were: 13.69 ± 1.06 (CS/HAp), 12.82 ± 4.10 (CS/TCP-α), 13.87 ± 2.9 (CS/HAp/TCP-α), and 15.55 ± 0.56 (CS/FAp). Consequently, various benzophenone concentrations were added to CS/HAp formulations to determine their effect on the degradation rate. Based on the mechanical properties and degradation profile of CS/HAp, it was found that 5 µL of 0.1% (*w/v*) benzophenone resulted in the highest degradation rate at eight weeks (54.48% degraded), while maintaining compressive strength between (4.04 ± 1.49 to 10.17 ± 4.78 MPa) during degradation testing. These results indicate that incorporating bioceramics with a suitable photoinitiator concentration can tailor the biodegradability and load-bearing capacity of the scaffolds.

## 1. Introduction

Bone tissue defects are a rising global concern and are one of the leading causes of morbidity and disability, especially in elderly patients. Normal fractures typically heal within 6–8 weeks in healthy adults, but in 5–13% of cases, the bone does not heal properly and can lead to delayed or non-union, which is classified as the absence of bone healing signs three months post-trauma [1,2,3,4,5,6]. A critical-sized bone defect is a defect that exceeds the natural capacity of bone healing, and these are the major cause of the non-unions, which necessitate a planned reconstruction and secondary surgery to implement and add to the patient’s burden and overall cost of the treatment [7,8]. Fracture repair is a global challenge, with about 3.5 million new bone fractures recorded in European Union citizens, involving hip fractures, vertebral fractures, forearm fractures, and other fractures [9]. The non-union rates in the United States is 1.9–10%, while in the United Kingdom (UK), 5–10% out of approximately 850,000 new fracture cases were identified or 0.02% in 100,000 population [10,11].

Autografts harvested from the patient’s own non-essential bone stock are considered the gold standard in treating fractures. Autografts account for 2.2 million transplantations in orthopaedic and dentistry repair worldwide due to their high success rate of 80–90% [12,13,14,15,16,17]. While bone grafts can be donated (allograft) to support and supplement the limited supply of autografts, these treatments require using a considerable amount of the bone grafts with a higher potential risk of disease transmission or even being rejected by the recipient’s body [8,18,19]. Therefore, an engineered bone scaffold is an attractive alternative treatment for bone fractures replacing these conventional autologous and allogenic treatment options. Scientists are now focusing on fabricating bone scaffolds that can mimic specific cellular responses at the molecular level by using various natural or synthetic biomaterials and also combinations of these, such as collagen, gelatin, silk fibroin, chitosan, alginate, polycaprolactone (PCL), polylactic acid (PLA) and poly (lactic-co-glycolic acid) (PLGA) [8,12,20,21,22,23,24,25].

Additionally, the bone scaffolds are tailored to have compulsory characteristics in line with the diamond concept of bone graft substitutes through the combination of osteoconductive materials, osteoinductive growth factor, osteogenic cells, and adequate mechanical stability [26,27,28]. The development of scaffolds infiltrated with osteogenic factors released in an extended period is a promising acellular strategy to promote bone formation. The first commercially available and FDA-approved growth factor delivery system for bone healing treatment is known as Medtronic Infuse^®^, which incorporates recombinant bone morphogenetic protein-2 (rhBMP-2) in a collagen sponge carrier [29]. However, this system requires intra-operative preparation and is associated with high burst release that can lead to unwanted adverse effects, including higher rates of implant displacement, subsidence, infection, ectopic bone formation, osteolysis, and thus may result in ineffective treatment of non-unions [30,31,32,33].

The scaffold biodegradation profile plays an essential role in releasing a loaded drug through customising these scaffolds to degrade at a similar rate to the bone ingrowth, where the newly formed bone gradually replaces the scaffolds in an osteotransductive manner, thus obtaining a suitable drug release mechanism [5,34,35]. Since chitosan (CS) can undergo enzymatic degradation in vivo due to its degradable glycosidic chains and the degradation products can be digested naturally after entering the bodies’ metabolic cycle, a plethora of studies have been carried out in fabricating bone scaffolds involving this biopolymer [17,36,37,38,39]. CS is also popular since it is derived from natural sources (typically the exoskeleton of crustaceans and insects), it is biocompatible, has antimicrobial properties, and is non-toxic as well as osteoconductive, making it a versatile biomaterial [17,39,40]. 

The in vivo performances of CS may be different depending on its deacetylation degree, molecular weight and also functionalisation with the other chemical groups, such as trimethylated chitosan, which can be tailored based on the specific necessities [41]. It was reported that high deacetylated CS will degrade slowly in vivo and may reach several months before being completely degraded, while rapid degradation can be achieved by using a low deacetylated CS [30]. Moreover, the performance of CS in BTE is restricted by its being insoluble in neutral pH and its insufficient mechanical stability, where De Witte et al. (2018) proposed that a bone scaffold should possess a compressive strength between 2–12 MPa and an elastic modulus of 0.5–1 GPa [42]. However, chemical modifications utilising the crosslinking of CS to another material allow the possibility to introduce different functional groups to the CS chain and thus altering the properties of the composite in achieving the desired scaffold features [43]. Previously, it was reported that a CS/HAp scaffold fabricated using novel ultraviolet (UV)-crosslinking procedures failed to degrade after ten weeks in the simulated body fluid, which might disturb the bone turnover process in vivo [5].

Hence, researchers have examined the effect of combining CS with osteoinductive bioceramics, including hydroxyapatite, tricalcium phosphate and bioactive glass, to overcome these limitations [25,44]. These osteoconductive ceramics have biologically similar inorganic components to the natural bone and bone-bonding properties, enabling the regenerated bone tissue to form a chemical bond with the scaffold surfaces [36,45]. Furthermore, the addition of these bioceramics into scaffold formulations was previously investigated to increase the tensile and compressive strength. These biomaterials are also proven to accelerate tissue healing without requiring further surgical procedures after implanting polymeric scaffolds, as they can deteriorate naturally or be wholly integrated with the newly formed tissue once implanted [41]. 

**Figure 1:**
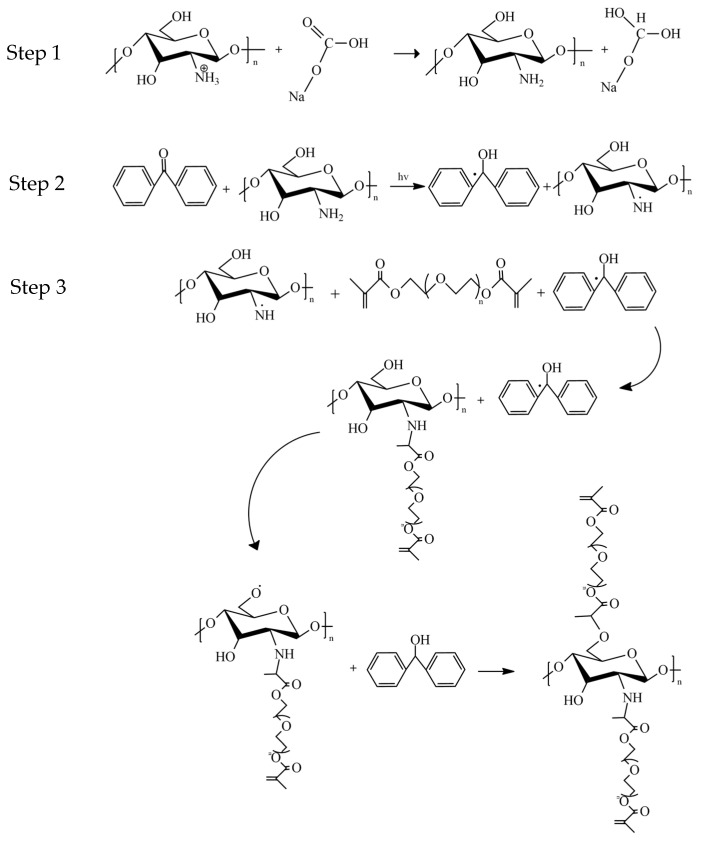
An extended photo crosslinking postulated. The protonated chitosan was neutralised with sodium bicarbonate solution (Step 1) and reacted in UV light in the presence of benzophenone producing a radical (Step 2). Further UV irradiation produces another available radical, leading to a stronger crosslinking (Step 3).

Based on these findings, it is hypothesised that it will be possible to create a CS-ceramic scaffold with suitable mechanical and swelling properties using one-step hydrogen abstracting free radical initiation process to crosslink the CS matrix. This approach will address the extended crosslinking reaction (Figure 1) of CS scaffolds with several types of bioactive ceramics incorporation and different concentrations of photoinitiator application that was not yet investigated. It is hypothesised that this will lead to tuneable bioresorption rates while maintaining strong crosslinking and high load-bearing capacity.

## 2. Results and Discussion

Prepared scaffold composites were assessed to determine the crosslinking efficiency, mechanical strength and chemical composition to optimise scaffold strength and degradation properties. 

### 2.1. Analysis of Crosslinkage Formation Following UV-Photocrosslinking Procedure

Swelling studies were conducted in 1% (*v/v*) acetic acid solution due to chitosan’s insolubility in water. As such, the swollen samples in 1% (*v/v*) acetic acid would dissolve if not crosslinked. 

**Figure 2:**
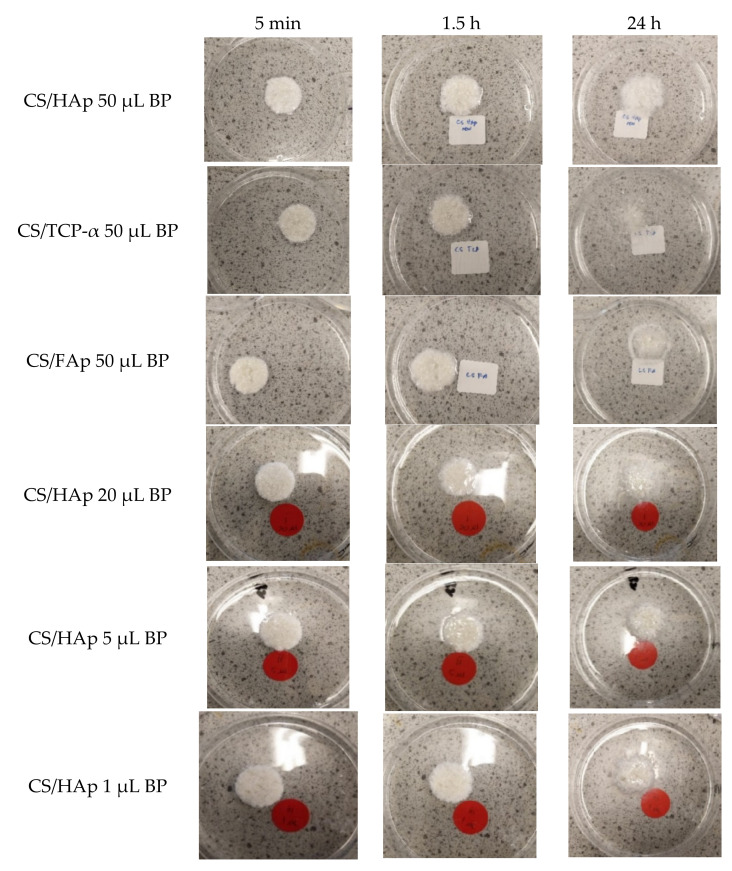
Photographs of CS scaffolds with various ceramics and BP content which were placed in 1% *v/v* acetic acid solution in the water acting as a solvent for CS. Minimal swelling or dissolution was observed for up to 24 h indicating the success of the crosslinking reaction.

The scaffolds were visually inspected following the submerging in 1% (*v/v*) acetic acid at 5 min, 1.5 and 24 h (Figure 2). Among the various ceramic compositions, it was found that CS/TCP-α and CS/FAp swelled the most in the acetic acid in 24 h, leading to difficulty during handling. Upon varying the BP content, CS/HAp scaffolds with 5 µL of benzophenone were observed to remain intact during handling compared to CS/HAp/20 µL BP and CS/HAp/1 µL BP. 

The gel fraction (GF) is a measure of the percentage of unreacted polymer components which had leached from the scaffold during testing [46,47]. GF in acidic conditions (Table 1) indicates partial crosslinking of the scaffold [41,48,49]. CS/TCP-α exhibited the highest value among the various ceramics scaffolds with a value of 67.1465 ± 7.93 followed by CS/HAp (55.437 ± 6.37) and CS/FAp (49.0943 ± 4.01). Evaluating the scaffolds with different BP contents, the CS/HAp/BP 5 µL recorded the lowest gel fractions in acetic acid of 51.1598 ± 4.16 compared to 56.5475 ± 2.46 for CS/HAp/10 µL BP and 53.4653 ± 4.79 for CS/HAp/20 µL BP. The lowest gel fraction value of CS/HAp/5 µL BP subsequent to the submerging in acetic acid is accepted as an adequately crosslinked scaffold considering the least photoinitiator used since samples did not dissolve after 24 h in the acetic acid solution, indicating that crosslinking had occurred [25,50,51,52]. On top of that, this low BP usage is more desirable to avoid any hazard of unreacted BP to the human body [52,53,54]. 

Recently, Nokoorani et al. (2021) investigated the effect of different concentrations of allantoin in chitosan/gelatin scaffolds for wound healing application and recorded gel fraction in double distilled water with values of 86–89% upon utilising 1-Ethyl-3-[3-dimethylaminopropyl] carbodiimide hydrochloride (EDC) as their crosslinker [47]. As chitosan is insoluble in water, these values are not comparable to results in the acetic acid and as such tests were repeated in PBS to determine comparable data and to replicate the human in vivo environment (Table 2). GF in PBS indicated that CS/FAp had the lowest gel fraction in PBS (94.55 ± 1.03), while similar values were obtained for CS/HAp and CS/TCP-α scaffolds at 99.28 ± 0.59 and 99.72 ± 0.88, respectively. In addition, no significant differences were shown in GF in PBS for scaffolds with various BP content, exhibiting the values of 97.40 ± 1.86, 96.83 ± 0.27 and 96.34 ± 0.18 for CS/HAp/5 µL BP, CS/HAp/10 µL BP and CS/HAp/20 µL BP, respectively. These results demonstrate the highest GF in PBS for CS/HAp/5 µL BP with only ± 3% unreacted materials being washed away compared to the other two BP concentrations. These results were >10% higher than previously reported data from swelling CS scaffolds in ddH2O. 

Moreover, it was evident that the CS/FAp-containing scaffolds recorded the highest values in EWC, WU, and swelling percentage showing the weak crosslinking degree of the scaffolds. EWC of the scaffolds were shown to be 59.29 ± 0.95 for CS/TCP-α, 65.74 ± 0.79 for CS/HAp and 87.71 ± 1.92 for CS/FAp scaffolds. Meanwhile, the EWC for the scaffolds with different BP contents were 63.61 ± 0.43 (CS/HAp/5 µL BP), 64.60 ± 0.5 (CS/HAp/10 µL BP) and 64.47 ± 0.08 (CS/HAp/20 µL BP), indicating that their swelling ability was around a similar level despite the varied photoinitiator amount. While the EWC of human bone is about 15–25%, this shows the importance of water in bones since it is the main factor affecting the mechanical behaviour of the bones [55,56]. As such, a lower EWC would be desirable. 

In addition, the swelling test recorded the water uptake (WU) capacity of 145.74 ± 5.68, 191.97 ± 6.84, and 726.65 ± 127.2 for CS/TCP-α CS/HAp and CS/FAp scaffolds, respectively, while CS/HAp/5 µL BP, CS/HAp/10 µL BP and CS/HAp/20 µL BP recorded WU values of 174.85 ± 3.28, 182.51 ± 4.04 and 181.43 ± 0.63, respectively, showing a reduced WU for CS/HAp/5 µL BP. Although water uptake is essential for nutrient transport and gas interchange, swelling under physiological conditions should be controlled to avoid excessive degradation from the diffusion of water into weakly crosslinked scaffolds and thus further causing the loss of mechanical integrity and compressive stresses to the cellular environment [36,57,58,59]. A similar trend was also shown in the percentage of swelling, with values of 826.65 ± 127.22 (CS/FAp), 291.97 ± 6.84 (CS/HAp), 245.74 ± 5.68 (CS/TCP-α), while different BP content-scaffolds presented the swelling percentage of 274.85 ± 3.28 (CS/HAp/5 µL BP), 282.51 ± 4.04 (CS/HAp/10 µL BP) and 281.43 ± 0.63 (CS/HAp/20 µL BP). Higher and faster swelling behaviour in PBS of pH 7.4 corresponding to blood pH indicates the scaffolds’ hydrophilicity and porosity [48,60]. Based on the swelling data, it appears that the relative porosity of scaffolds were: CS/FAp > CS/HAp > CS/TCP-α and CS/HAp/20 µL BP > CS/HAp/10 µL BP > CS/HAp/5 µL BP. It has been previously reported that crosslinking reactions are normally influenced by the type of crosslinker and its concentration as well as the reaction time [43,52,61]. These results validated that a high crosslinking density can be achieved even with a low BP content due to the reversible BP excitation, enabling the diradicals to revert to the ground state and continue to react with the favourable CH- group, thus ensuring an efficient covalent bonding [62]. However, since the cross-conjugation took place using UV light, free radicals may be present in the scaffold. As such a ROS test should be conducted to ensure radicals are not present in the final construct.

### 2.2. Fourier-Transform Infrared Spectroscopy Analysis

**Figure 3:**
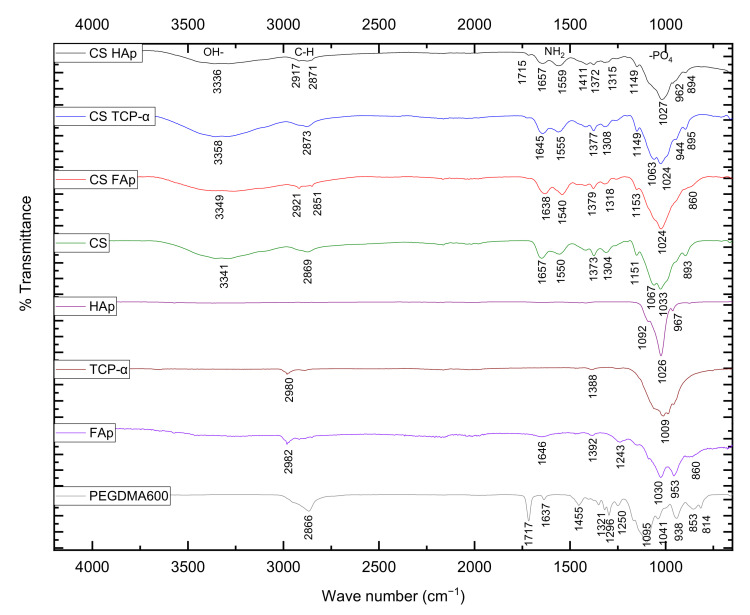
FTIR spectrum of individual components and different CS/ceramic scaffolds following the crosslinking reaction.

The chemical compositions of the fabricated scaffolds were analysed using FTIR. In this work, chitosan exhibited peaks corresponding to N-H stretching and OH- peaks at 3336–3358 cm^−1^, asymmetrical C-H stretch of -CH_2_ at 2869–2921 cm^−1^, C=O stretching of amide I at 1638–1657 cm^−1^, the N–H deformation of amide II at 1540–1559 cm^−1^, C–CH_3_ band at 1372 cm^−1^ and the saccharide C–O–C stretching at 1149–1153 and 1024–1027 cm^−1^, which correspond to reported values in the literature (Figure 3) [63,64,65,66]. 

HAp powder presented characteristic peaks corresponding to PO_4_^3−^ at 1026 and 1092 cm^−1^, while scaffolds containing HAp exhibited PO_4_^3−^ peaks at 1027 and 1149 cm^−1^, with phosphate symmetrical stretching vibration at 962 cm^−1^ and CO_3_^2−^ groups at 1411–1657 cm^−1^. Referring to the literatures, hydroxyapatite is commonly identified through the presence of symmetrical phosphate stretching (950–962 cm^−1^), orthophosphate asymmetrical stretching (1029, 1089, 980–1100 cm^−1^), carbonate groups (1670–1420, 1418, 1471 cm^−1^) as well as hydroxyl group stretching bands (3372–3348, 3570, 3575 cm^−1^) [63,67,68,69]. 

TCP-α presented phosphate peak at 1009 cm^−1^ and the presence of TCP-α scaffolds was indicated by PO_4_^3−^ peaks at 944, 1024, 1063 and 1149 cm^−1^ and CO_3_^2−^-region at 895 cm^−1^, validating the structural characteristics of the calcium phosphate ceramics. TCP-α is characterised by its main PO_4_^3−^ bands of v_3_ anti-symmetric P-O stretching at 1107, 1058, 1039, 1013, 1022 and 984 cm^−1^, as well as v_1_ symmetric P-O stretching at 935–938, 954–959 cm^−1^ [70,71,72,73,74]. FAp is characterised by its v_3_ phosphate ions at 960, 1020–1026, and 562 cm^−1^, where scaffolds produced in this work which contained FAp demonstrated the presence of PO_4_^3−^ peak at 1024 cm^−1^, which is in agreement with the values reported in the literature [75,76,77].

PEG600DMA contains two different unsaturated bonds at both ends of its repeated unit. These peaks are represented on FTIR spectra at 1637–1650 cm^−1^ for C=C and 1720–1760 cm^−1^ for C=O [78,79,80,81]. In this work, corresponding peaks were observed at 1637 cm^−1^ for C=C and 1717 cm^−1^ for C=O. Following the UV reaction, these peaks were observed at 1638–1657 cm^−1^ for C=C and 1715 cm^−1^ for C=O with reduced intensity.

**Figure 4:**
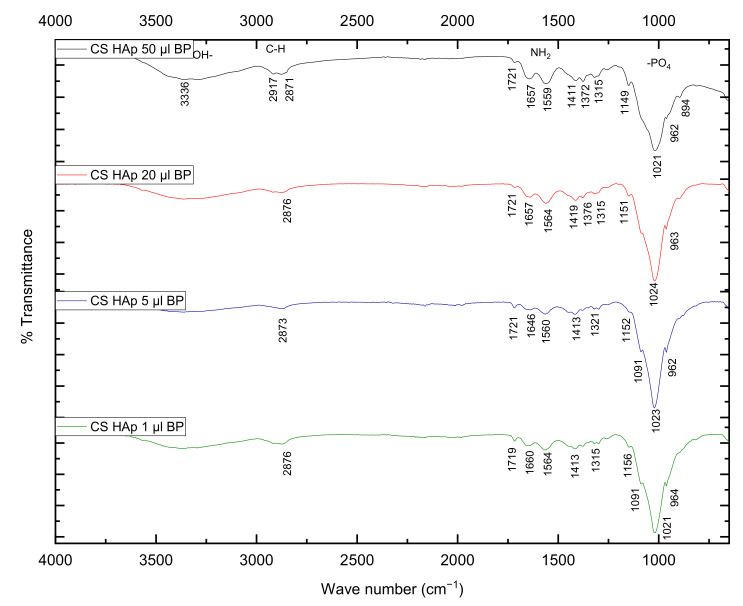
FTIR spectrum of CS/HAp scaffolds incorporating 50, 20, 5 and 1 µL BP at 0.1% *w/v*.

The FTIR spectrum of the CS/HAp scaffolds with different BP contents (50, 20, 5 and 1 µL of 0.1% *w/v* benzophenone) in Figure 4 shows that the CS/HAp scaffolds retained the structural properties with no major peaks shifting following the crosslinking reaction as outlined earlier in this section. However, with varying concentrations of BP, it was observed that the (C=C) peaks appeared to reduce with decreases in BP concentration. This peak reduction indicates breakage of the bond during crosslinking, which may have resulted in crosslinking of the chitosan structure as supported by swelling studies.

### 2.3. Mechanical Assessment

**Figure 5:**
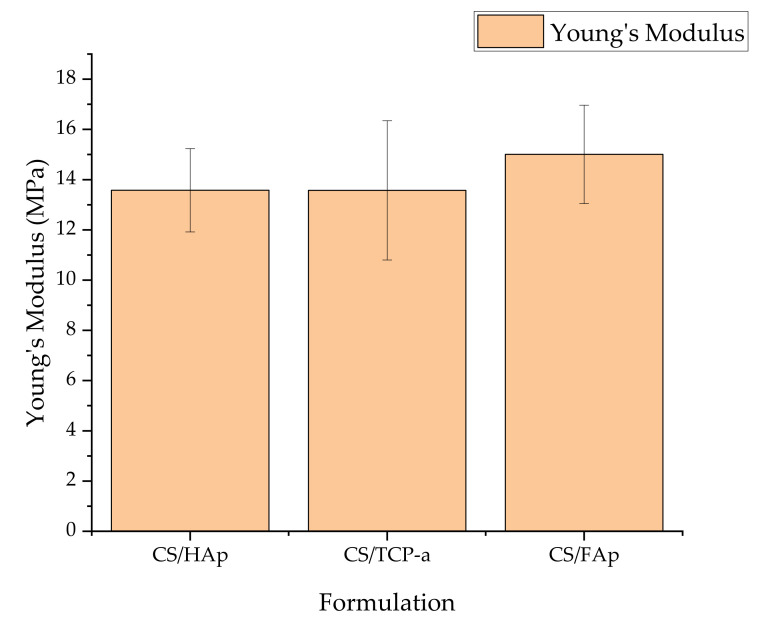
The compressive strength of the chitosan scaffolds with different bioceramics recorded similar values achieving a strength above 12 MPa.

Compression testing was performed to assess the mechanical performance of the tissue engineering scaffolds. Healthy cortical bone has a strength of 100–150 MPa. However, most autografts consist of cancellous bone, which has a strength of 1.5–38 MPa [25,82,83,84]. It was found that the currently formulated scaffolds had Young’s modulus values of 13.69 ± 1.06 MPa (CS/HAp), 12.82 ± 4.10 MPa (CS/TCP-α), and 15.55 ± 0.56 MPa (CS/FAp) at 60% strain (Figure 5), which falls into the range of cancellous bone [85]. Similarly, Borkowski et al. (2021) reported that their FAp/ß-1,3-glucan scaffolds achieved a compressive strength of 11.55 MPa, which is higher than their HAp/ ß-1,3-glucan scaffolds of 6.57 MPa. Since ß-1,3-glucan is also a polysaccharide equivalent to chitosan, their compressive strength values are relevant to the scaffolds produced in this work [86]. These results also validated that the crosslinking of chitosan polysaccharides does enhance the mechanical properties of the scaffold products [61].

**Figure 6:**
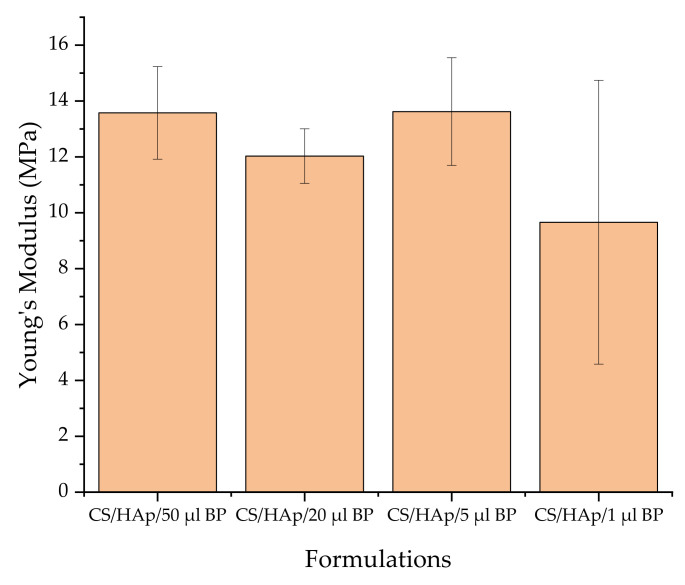
The Young’s modulus values of the chitosan scaffolds with different BP contents present the lowest strength in the least BP volume.

Alterations in the photoinitiator concentration in the scaffold formulations did not have a significant effect on the compressive strength of the CS/HAp scaffolds (Figure 6), where values of 12.03 ± 0.98 (CS/HAp/20 µL BP), 13.62 ± 1.93 (CS/HAp/5 µL BP) and 10.75 ± 3.93 MPa (CS/HAp/1 µL BP) were recorded (*p* > 0.05, for all comparisons). These values are higher than values reported in the literature for bone tissue engineering scaffolds, where the compressive modulus of chitosan/HAp-based composites and also aneroin/HAp-3D complex construct were between 4–6 MPa at 60% stain and 6.42 MPa at 40% strain, respectively [5,87]. Additionally, the recorded compressive strength values are also higher than the values reported by Zhang et al. (2019) from the silk fibroin (SF), carboxymethyl chitosan (CMCS), cellulose nanocrystals (CNCs) and strontium substituted hydroxyapatite (Sr-HAp) scaffold combinations, ranging from 22.91 ± 3.24 KPa to 78.55 ± 5.04 KPa [60].

The mechanical strength of the scaffolds is important for the recovery of two primary factors in bone healing: the load-bearing capacity and bone strength. Mechanical stimulation on the cellular level in the healing area will contribute to normal bone repair and regeneration in three stages of healing: mesenchymal stem cells proliferation in the early inflammatory phase, soft callus/non-mineralised cartilage in the reparative phase and the hard callus reconstitution in the remodelling phase [16,42,64,88,89,90]. Among these stages, it was found by Fu et al. (2022) that changes in mechanical stimulus can more easily manipulate the early healing process than the later ones due to the micro-motion following the initial flexible fixation [91,92,93,94,95,96].

### 2.4. In Vitro Biodegradation Assessment

The biodegradability of a scaffold is another vital characteristic of an engineered bone scaffold. The ideal scaffold is postulated to be able to have a degradation rate similar to the rate of the new bone formation to promote ideal bone healing [15,48,50,78,83,97,98]. In this work, biodegradability in the presence of simulated body fluid (SBF) was monitored to achieve the desired degradation rate of eight weeks, which is equivalent to the bone healing time frame for healthy bone [27,99,100,101]. 

**Figure 7:**
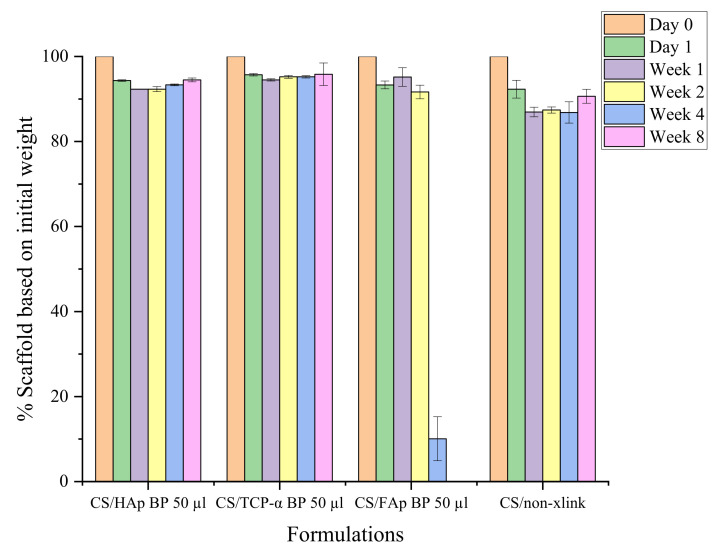
The degradation profile of the chitosan scaffolds in simulated body fluid over eight weeks shows a similar degradation rate for chitosan scaffolds with HAp, TCP-α and FAp ceramics.

**Figure 8:**
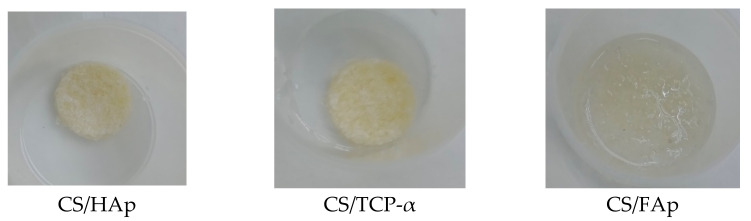
The scaffolds’ physical condition following submerging in SBF for two weeks, where the swelling of chitosan was observed the most in CS/FAp.

After one week in the SBF, it was shown that the non-crosslinked chitosan scaffolds had lost 7.69%, which was higher than the other scaffolds tested (Figure 7). This weight loss is postulated to result from the absence of UV photocrosslinking that failed to bind the biomaterials together or make a weak network, thus leading to earlier biodegradation [60]. It was observed from the weight-loss trend that there was a slight increase in weight at the end of the test on week eight for both CS/HAp and CS/TCP-α, which could be due to calcium phosphate salt deposition from SBF increasing the remaining scaffold weight [102,103,104]. However, a major 81.58% weight loss was recorded for CS/FAp scaffolds between weeks 2 and 4. This high degradation rate is unlikely for fluorapatite since it was reported that FAp possesses higher resistance towards degradation in physiological conditions compared to HAp upon the insertion of F- ions into OH- groups [75,105,106]. Nevertheless, this phenomenon might be due to the higher polarity of HAp compared to FAp. As such, HAp binds more easily to the polar groups of chitosan, thereby binding the structure together and preventing high levels of swelling and degradation. Conversely, the chitosan can swell more easily in CS/FAp (Figure 8) due to the electrostatic repulsion, allowing fluid ingression, which leads to an increase in hydrolytic degradation [73].

**Figure 9:**
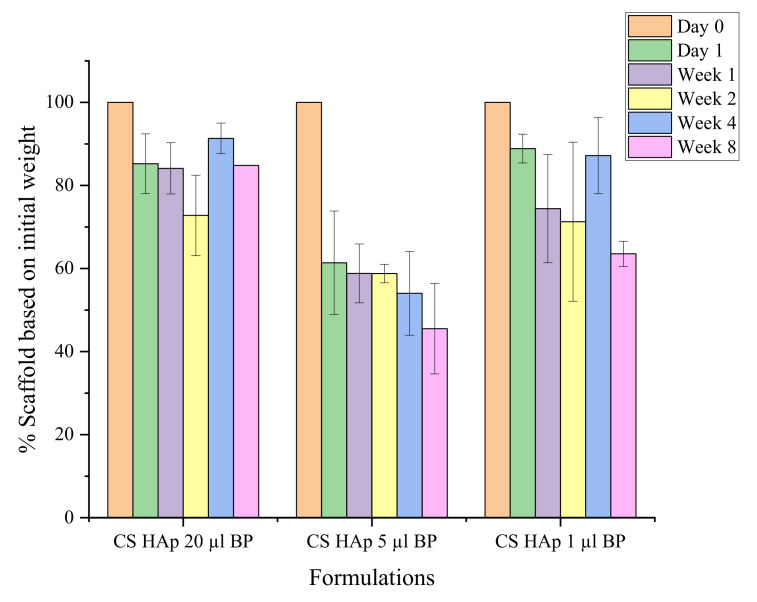
The degradation profile of CS/HAp scaffolds with different benzophenone content in SBF, where CS/HAp/5 µL BP presented a stable degradation rate for eight weeks.

Additionally, BP loading also affected the degradation rate of the scaffold (Figure 9). Degradation data indicated that an increased degradation was observed in CS/HAp scaffolds with reduced BP contents. Gradual and stable degradation can be observed in the CS/HAp/5 µL 0.1% *w/v* BP scaffold profile, where 54.48 (±10.89)% weight degraded after eight weeks in SBF. The scaffolds were then left to continue degrading until week 12 and were observed to lose their integrity during handling. As such no measurement was possible at this stage, thus establishing that this representative high-strength scaffold can induce osteotransduction within the desired timeframe [34,39,107]. This degradation period corresponds to a report by Turnbull et al. (2017) documenting that a porous ß-dicalcium silicate (ß-Ca2SiO4) scaffold was aimed at bone healing applications [108]. The loss of the scaffold integrity within this period will enable the permeation of bone healing molecules and mechanisms within the bone defect facilitating the bone regeneration process [109]. In general, the changes in photoinitiator concentration are indeed leading to the tuneability of the degradation rate, pore size and porosity of the scaffolds [8].

#### Mechanical Stability during Biodegradation

Since scaffolds degraded slowly over the increasing incubation period, the scaffolds’ strength was also presupposed to decrease over time [110]. The compression test was performed on the scaffolds following the degradation time points to evaluate the strength of the scaffolds while disintegrating in the SBF solution. 

**Figure 10:**
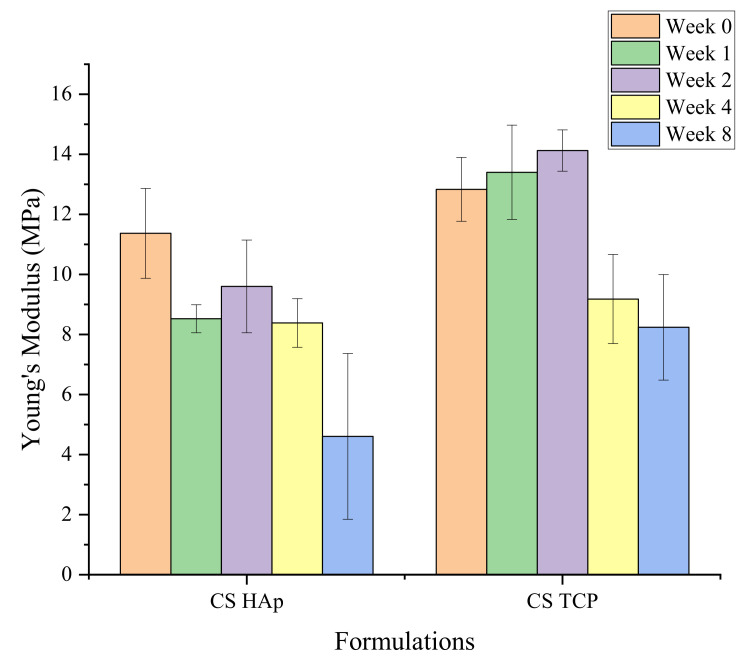
The compressive strength profile of the scaffolds while degrading in SBF for CS/HAp and CS/TCP-α, showing a reduction in the scaffold strengths over the eight weeks.

The compression test result (Figure 10) shows that the strength of the scaffolds for both formulations decreased while degrading over eight weeks. The strength of the CS/HAp scaffold decreased from 13.69 (±1.06) MPa after one day to 5.46 (±2.47) MPa after eight weeks, while CS/TCP scaffolds had reduced the strength from 12.82 (±4.1) MPa on day one to 8.24 (±1.76) MPa on week eight of the degradation test with significant difference recorded for both formulations (*p* < 0.05, for both comparisons). The decreasing trend of Young’s modulus values obtained in this degradation procedure could indicate that the bonds and linkage between the materials were broken gradually, thus validating that biodegradation of the materials had occurred [111]. 

**Figure 11:**
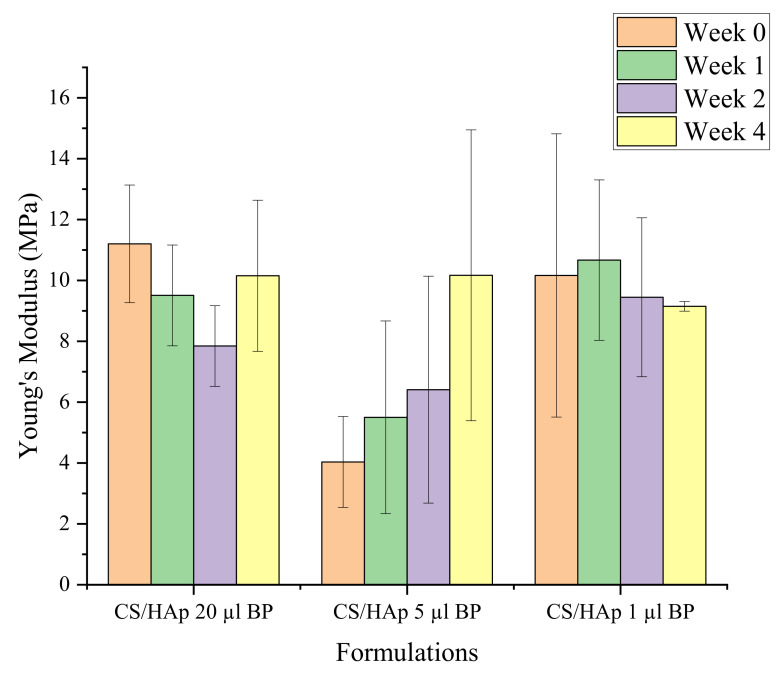
The compressive strength profile of the CS/HAp scaffolds with different BP contents while degrading in SBF for eight weeks. An increase in strengths might be indicative of the remaining strong crosslinked chains following the initial disintegration of the weaker chains.

The compressive strength of the scaffolds fabricated with various BP content while degrading was also tested (Figure 11). It was found that these BP volumes which were lower than the previous 50 µL had lost their integrity during handling after four weeks in the SBF, and were thus unable to be tested after week 4. The CS/HAp scaffolds with 20 µL 0.1% *w/v* benzophenone showed a reduction in strength from the initial 11.20 MPa to 7.85 MPa after week 2 before increasing to 10.15 MPa in week 4. Interestingly, the compressive strength of CS/HAp/5 µL BP was observed to increase over the four weeks in the SBF. However, no significant difference was observed, illustrating that the scaffolds had maintained their strength for four weeks prior to complete disintegration in week 8 (*p* > 0.05, for all comparisons). An increase in Young’s modulus values after the initial reduction observed could be explained by the polymeric structural breakdown process. In the beginning, the shorter polymer chain in the scaffold composite has broken down, leaving the longer chains behind. Thus, this could result in the greater strength of the scaffold before the breaking down of the longer chain takes place. This result was highly preferable as it was aimed to maintain the strength of the scaffold following the application in vivo [112].

### 2.5. Scanning Electron Microscopy and Energy Dispersive X-ray Spectrometry

**Figure 12:**
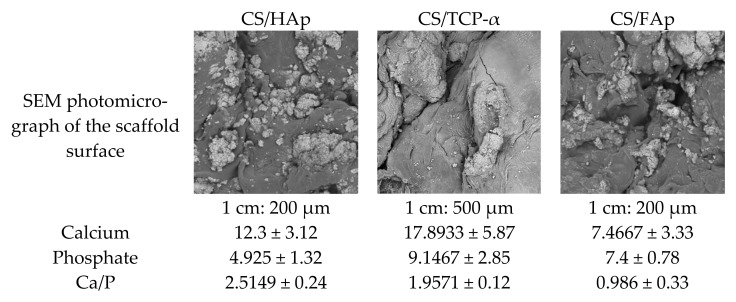
SEM-EDX analysis for the scaffolds showing the highest Ca/P values in CS/HAp followed by CS/TCP-α and CS/FAp.

Surface analysis was performed using SEM and the elemental compositions were confirmed using EDX. The main elements recorded for all scaffolds were Carbon (C), Oxygen (O) and Sodium (Na), which originated from the neutralised chitosan, while Calcium (Ca) and Phosphorus (P) represented the incorporated ceramics. In addition, the unique Fluorine (F) component present in CS/FAp scaffolds (0.9%) validated the fluorapatite prepared, although this value was less than that of the prepared FAp powder (2.5%). Chen et al. (2006) and Kimoto et al. (2011) previously validated the synthesised FAp through the presence of a fluorine peak in the EDX analysis compared to the absence of this peak in the HAp sample, thus showing the importance of fluorine in characterising FAp [113,114]. CS/HAp scaffolds presented a Ca/P ratio of 2.5149 ± 0.24, which is higher compared to the theoretical value for HAp alone and human bones of 1.66 and 2.2-1, respectively (Figure 12) [58,115,116,117,118,119,120,121,122]. It is hypothesised that this higher than the expected Ca/P ratio was due to the presence of unreacted calcium chloride residues in HAp [5,123]. Additionally, CS/TCP-α presented a Ca/P ratio of 1.9571 ± 0.12 which is higher than the TCP stoichiometric value (1.5) but lower than the previously reported value for TCP-ß (2.02). This result might be due to the mixtures of two types of TCP, namely TCP-α and TCP-ß. However, CS/FAp exhibited a Ca/P ratio of 0.986 ± 0.33 compared to its theoretical value (1.67) and previously reported Ca/P for FAp (1.4), thus showing the lesser mineralisation occurred [113,122,124,125]. 

In addition, the SEM photomicrographs also revealed the porosity of the fabricated scaffold composites, which was analysed by using ImageJ software. CS/HAp presented 3.68 ± 0.2% porosity, followed by CS/FAp (3.06 ± 0.2%), and CS/TCP-α (3.02 ± 0.1%), where these values are close to the reported porosity of the cortical bone (5–30%). While it was proposed that a scaffold should possess a porosity of >90% to facilitate optimum nutrients diffusion, this will compromise the mechanical strength and thus leading to failure of load-bearing support [8,126]. 

**Figure 13:**
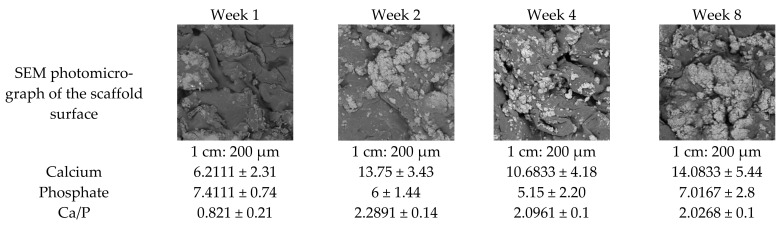
SEM-EDX analysis for the degraded CS/HAp scaffolds in weeks 1 to 8, showing a low Ca/P value in week 1 before increasing in week 2 and decreasing gradually to week 8.

The surface and elemental analyses were also conducted on the degrading CS/HAp scaffolds to observe the Ca/P ratio representing the apatite formation in SBF (Figure 13). The Ca/P ratio of the scaffold during the first week of degradation was observed to be the lowest (0.821 ± 0.21) compared to the later time points. This supports the theory that unreacted calcium chloride was present in the HAp. Due to its relatively high solubility, these calcium compounds had dissolved, leading to a reduction in the Ca/P ratio at one week. Subsequently, the Ca/P ratio increased again due to mineral deposition from the SBF as reported by Zhang et al., (2019), Shemshad et al., (2019) and Wu et al., (2020) [60,127,128]. The ratio continued to decrease during the degradation study which appears to be led by an observed increase in P levels supporting the mineral deposition theory. Previously, pig bone-derived HAp was observed to reduce the Ca/P ratio after being soaked in SBF, indicating the gradual deconstruction of the ceramic in SBF as well as the elimination of organic moieties from the samples, which might as well explain the scaffolds’ behaviour in this work [129].

## 3. Conclusions

Developing a biodegradable bone scaffold with sufficient mechanical properties and can provide osteoconductive features is crucial in this tissue engineering field. The present study demonstrated an improved chemical crosslinking method of a chitosan-based scaffold with various bioceramics and photoinitiator alterations. The swelling test in acidic conditions presented a gel fraction of more than 50%, demonstrating crosslinking was achieved. The FTIR showed the scaffold characteristics for both chitosan and ceramics following crosslinking, where a reduction in C=C peaks were recorded with decreasing BP content. Moreover, the surface morphology was confirmed through SEM-EDX analysis. The compression testing validated the mechanical performance of the scaffolds achieving 12–15 MPa, which was sustained to at least 10 MPa during degradation recorded over eight weeks in SBF. The scaffold mineralisation in SBF was monitored semi-quantitatively using SEM-EDX, where an increase in Ca/P ratio was recorded from week 1 to week 2 before gradually reduced from week 2 to week 8. 

CS/HAp with 5 µL of 0.1% *w/v* benzophenone scaffold formulation is proposed for future investigation as a biomimetic bone scaffold candidate since it was found promising mechanical properties while degrading in addition to favourable swelling and gel fraction characteristics. Ultimately, the scaffold fabrication method presented is hypothesised to provide better control of the covalent grafting of active ingredients between the scaffold structure and its release in vitro and later in vivo.

## 4. Materials and Methods

Chitosan (high MW), hydroxyapatite, ethanol ≥ 99.8%, and sodium fluoride ≥ 99% were obtained from Sigma Aldrich. Sodium bicarbonate 99.5% was purchased from Acros Organics (Fisher Scientific UK Ltd., Loughborough, UK), poly(ethylene glycol) (600) dimethacrylate was obtained from Polysciences Inc. (Polysciences Europe GmbH, Germany) and benzophenone, 99% (A10739.30) was purchased from Alfa Aesar (Thermo Fisher (Kandel) GmbH). All materials were used as received.

### 4.1. Fabrication of Chitosan-Based Bone Regeneration Scaffold

A chitosan-based scaffold was prepared by dissolving 1.5 g of high MW chitosan powder in 12.5 mL of 1% (*v/v*) acetic acid, yielding a 12% (*w/v*) paste. The paste was left on the bench for an hour, allowing a protonation reaction to occur before being neutralised in 0.1 M sodium bicarbonate solution for 10 min. It was then pressed between filter papers to remove excess sodium bicarbonate solution. Subsequently, 100 µL of PEGDMA600 and 50 µL of 0.1% (*w/v*) benzophenone in ethanol were consecutively added to the paste and mixed well.

The chitosan paste was then transferred into silicone moulds to make 20 mm circular tablets and subjected to an ultraviolet (UV) crosslinking process using a UV curing system (Dr. Gröbel UV-Electronik GmbH, Opsytec Dr. Gröbel, Ettlingen, Germany) under 20 UV lamps with a spectral range between 315–400 nm and at the average intensity of 10–13.5 mW cm^2^ for 40 min. All the samples were flipped over mid-process. This crosslinking time was determined experimentally in the previous investigations to be sufficient to cure the composites [5,130,131].

#### 4.1.1. Incorporation of Various Bioceramic Compositions

Chitosan paste was prepared as per Section 4.1. Following the addition of benzophenone, various ratios and combinations of scaffold formulations were made by adding HAp or TCP-α into chitosan paste with ratios as shown in (Table 3). After mixing, the sample pastes were placed in the silicon moulds and cured as outlined in Section 4.1.

Subsequently, fluorapatite was prepared from the chemical substitution of hydroxyapatite and sodium fluoride before incorporating into the chitosan-based scaffolds. A solution of 10 mol/L phosphoric acid (H_3_PO_4_) was prepared by making up 6.85 mL H_3_PO_4_ with water in a 10 mL volumetric flask. Subsequently, 3.2 mL of the acid solution prepared was added to 4.6 g of sodium fluoride (NaF). The mixture was subjected to magnetic stirring until all the NaF was dissolved. Once all the NaF was dissolved, the solution was added gradually to 5 g of hydroxyapatite powder until mixed thoroughly. The fluorapatite powder was finally obtained at the end of the process and was assessed using scanning electron microscopy and energy dispersion X-ray (SEM-EDX) to confirm the elements present. This FAp powder was then added into chitosan paste similar to the HAp and TCP-α mixing method, making CS/FAp composite (1:1).

#### 4.1.2. Modification of Chitosan-Based Scaffold Grafting Properties through Various Photoinitiator Composition

Subsequently, to investigate the effect of the photoinitiator concentrations on the scaffold degradation profile, CS/HAp scaffolds were fabricated as outlined in Section 4.1.1 with different amounts of 0.1% (*w/v*) benzophenone (50, 20, 5 and 1 µL) (Table 4) and followed by UV curing as mentioned in Section 4.1.

### 4.2. Crosslinking Test in 1% Acetic Acid

The crosslinkage between the materials post-UV curing process was qualitatively assessed by submerging the scaffolds in 1% (*v/v*) acetic acid for five minutes, 1.5 h and 24 h.

### 4.3. Swelling Behaviour

The scaffold fluid uptake under physiological conditions was evaluated through swelling studies on the crosslinked scaffolds (*n* = 3) with different bioceramic combinations (CS/HAp, CS/TCP-α and CS/FAp) and also the reduced photoinitiator (CS/HAp with 20, 10 and 5 µL of 0.1% *w/w* benzophenone).

The scaffolds were dried in the vacuum oven at 37 °C and 70 mbar for 72 h and recorded as the dry weight, w_d_. The scaffolds were then submerged in 1% acetic acid for 48 h before drying them again in the vacuum oven for 72 h in order to assess the effectiveness of the crosslinking reaction. The final equilibrium dry weight was recorded and denoted as w_ef_ before calculating the gel fraction in acetic acid (GF_AA_) by using the following formula:

$${\mathrm{GF}}_{\mathrm{AA}}=\frac{\mathrm{wef}}{\mathrm{wd}}\times 100$$

Subsequently, a new set of scaffolds as detailed above were further submerged in pH 7.4 phosphate-buffered saline (PBS) at ambient temperature for 48 h until the samples had reached swelling equilibrium. The samples were pat dried using filter papers, and the weight was recorded as w_s_. Subsequently, the scaffolds were dried again in the vacuum oven for 72 h until they reached equilibrium dry weight, w_e_. The swelling percentage, equilibrium water content (EWC), water uptake (WU) and gel fraction in PBS (GF_PBS_) of the scaffolds were calculated by using the formulas:

% Swelling = w_s_/w_d_ × 100

EWC = (w_s_ − w_d_)/w_s_ × 100

WU = (w_s_ − w_d_)/w_d_ × 100

GF_PBS_ = w_e_/w_d_ × 100

### 4.4. Fourier-Transform Infrared Spectroscopy (FTIR)

The linkage and structural properties of the scaffolds were analysed using FTIR spectroscopy on a Perkin-Elmer Spectrum One FTIR spectrometer fitted with a universal ATR sampling accessory. All samples were dried in a vacuum oven at 37 °C and 70 mbar prior to the tests to avoid the broad water peak from shadowing the significant signature peaks of the materials. All the tests were run using a spectral range of 4000–650 cm^−1^. Four scans per sample cycle were utilised with a resolution of 0.5 cm^−1^ at room temperature. Following the tests, the spectra obtained were analysed using Origin software.

### 4.5. Compression Test

The strength of the scaffolds was evaluated through compression testing using a Lloyd LRX tensometer (Lloyd Instruments Ltd., Hampshire, England, UK) with a 2.5 KN load cell in compression mode, and the results were analysed in NEXYGEN^™^ software. The samples were dried using a vacuum oven (Salvis Lab Vacucenter VC50, Rotkreuz, Schweiz) at 37 °C and 70 mbar for 24 h. Then, they were submerged in phosphate-buffered saline (PBS) solution for an hour before testing. Subsequently, the compression test was carried out at a rate of 0.5 mm/min and set to stop at 60% strain. The Young’s modulus values obtained from the software were analysed.

### 4.6. Degradation Test in Simulated Body Fluid

Simulated body fluid (SBF) was prepared using a previously documented method [132]. All chitosan scaffolds formulated were subjected to a biodegradation test in SBF for eight weeks. Before the test, the samples were dried in a vacuum oven at 37 °C and 70 mbar for 24 h. The dried weight of the samples was measured and noted as the dried weight before degradation, w_0_. The samples were then placed into small containers with a lid, and 5 mL of SBF were added to each container and kept in the oven (Gallenkamp Hotbox Oven with Fan Size 1, Sanyo, Weiss) at 37 °C under static conditions. The SBF solution for all samples was refreshed twice a week.

All samples were collected at weeks 0, 1, 2, 4 and 8 or until the samples were disintegrated during handling. The integrity was assessed during handling before being subjected to compression testing to evaluate their degradation profile and strength while degrading. Following the compression test, scaffolds were dried in the vacuum oven, and the weights were recorded as weight after degradation, w_1_. The degraded weights of all formulations were calculated using the following formula, where w_0_ represents the initial dry weight and w_1_ represents the final dry weight.

Degradation weight = (w_0_ − w_1_)/w_0_ × 100

### 4.7. Scanning Electron Microscopy and Energy Dispersive X-ray Spectrometry (SEM-EDX)

The scaffold surface morphology following the fabrication and biodegradation was assessed using a Mira SEM (TESCAN Performance in Nanospace) in backscattered electron (BSE) mode using magnifications ranging from 200–250×. Dried scaffolds were snapped to expose the cross-sectional regions before placing them on the stub giving both scaffold surface and cross-sectional view. Prior to imaging, the samples were gold sputter-coated using a Baltec SCD 005 for 110 s at 0.1 mBar vacuum. Energy Dispersive X-ray (EDX) scanning was conducted using an Oxford instruments detector to confirm the elemental composition of the composite components. 

### 4.8. Statistical Analysis

All statistical analysis in this document was performed using the Minitab software. Firstly, a descriptive statistic was performed to evaluate the mean and standard deviation between the samples. The significance between the two data groups was then tested using a student *t*-test. Subsequently, a one-way ANOVA was utilised to compare three or more parametric data groups, followed by a Tukey’s test to assess the significant changes in the data. Significance was achieved with data exhibiting a *p*-value less than 0.05 (*p* < 0.05).

## Figures and Tables

**Table 1:** Gel fraction values for scaffolds with varied ceramics and BP content in acidic conditions. The higher gel fraction values indicate a better linkage formed.

	Various Ceramic Compositions			Various BP Content		
Sample	CS/HAp	CS/TCP-α	CS/FAp	CS/HAp/20 µL BP	CS/HAp/10 µL BP	CS/HAp/5 µL BP
Gel fraction in 1% *v*/*v* acetic acid ± SD	55.437 ± 6.37	67.1465 ± 7.93	49.0943 ± 4.01	53.4653 ± 4.79	56.5475 ± 2.46	51.1598 ± 4.16

**Table 2:** Swelling studies of chitosan scaffolds with different ceramics (HAp, TCP-α and FAp) and benzophenone contents (20, 10 and 5 µL), comprising the EWC, WU, percentage of swelling and gel fraction in PBS.

Sample	Equilibrium Water Content, EWC ± SD	Water Uptake, WU ± SD	% Swelling ± SD	Gel Fraction PBS ± SD
CS/HAp	65.74 ± 0.79	191.97 ± 6.84	291.97 ± 6.84	99.28 ± 0.59
CS/TCP-α	59.29 ± 0.95	145.74 ± 5.68	245.74 ± 5.68	99.72 ± 0.88
CS/FAp	87.71 ± 1.92	726.65 ± 127.2	826.65 ± 127.22	94.55 ± 1.03
CS/HAp/20 µL BP	64.47 ± 0.08	181.43 ± 0.63	281.43 ± 0.63	96.34 ± 0.18
CS/HAp/10 µL BP	64.60 ± 0.5	182.51 ± 4.04	282.51 ± 4.04	96.83 ± 0.27
CS/HAp/5 µL BP	63.61 ± 0.43	174.85 ± 3.28	274.85 ± 3.28	97.40 ± 1.86

**Table 3:** Chitosan scaffold formulations with bioactive ceramics utilised for swelling, compression and biodegradation test.

	Weight (g)			Volume (µL)		Volume (mL)
	CS	HAp	TCP-α	BP	PEGDMA600	Acetic acid
1:1:0	1.5	1.5	0	50	100	12.5
1:0:1	1.5	0	1.5	50	100	12.5

Annotations: CS: chitosan; HAp: hydroxyapatite; TCP-α: tricalcium phosphate-α; BP: benzophenone; PEGDMA600: polyethylene glycol dimethaacrylate 600.

**Table 4:** Scaffold compositions with various benzophenone concentrations.

	Weight (g)		Volume (µL)		Volume (mL)
	CS	HAp	BP	PEGDMA600	Acetic acid
CS/HAp 1:1	1.5	1.5	20	100	12.5
	1.5	1.5	5	100	12.5
	1.5	1.5	1	100	12.5

Annotations: CS: chitosan; HAp: hydroxyapatite; BP: benzophenone; PEGDMA600: polyethylene glycol dimethaacrylate 600.
